# Promoting ecological hope as an antidote for eco-emotions and earth-related mental syndromes

**DOI:** 10.3389/fpsyg.2024.1471073

**Published:** 2024-10-23

**Authors:** Carlos Laranjeira, Helena Águeda Marujo, Zaida Charepe, Ana Querido

**Affiliations:** ^1^School of Health Sciences, Polytechnic University of Leiria, Leiria, Portugal; ^2^Centre for Innovative Care and Health Technology (ciTechCare), Polytechnic University of Leiria, Leiria, Portugal; ^3^Comprehensive Health Research Centre (CHRC), University of Évora, Évora, Portugal; ^4^Instituto Superior de Ciências Sociais e Políticas, Centro de Administração e Políticas Públicas (CAPP), Universidade de Lisboa, Lisboa, Portugal; ^5^Centre for Interdisciplinary Research in Health (CIIS), Universidade Católica Portuguesa, Lisboa, Portugal; ^6^Faculty of Health Sciences and Nursing, Universidade Católica Portuguesa, Lisboa, Portugal; ^7^Center for Health Technology and Services Research (CINTESIS), NursID, University of Porto, Porto, Portugal

**Keywords:** hope, climate change, ecology, mental health, sustainability, health vulnerability, uncertainty

## 1 Introduction

In the 21st century, the vulnerability of populations exacerbates global concerns related with climate issues and the need for sustainable development education. Extensive literature documents the negative impacts of climate change on mental health and wellbeing (Cianconi et al., 2020; Clayton, 2021; White et al., 2023). The challenges posed by climate change will intensify worldwide, particularly for vulnerable populations such as children, older people, refugees, and those with pre-existing health conditions. Mental health consequences will vary due to the close relationship between the climate crisis and individual vulnerability (White et al., 2023), which is influenced by three primary factors: exposure, sensitivity, and adaptive capability. At its core, the discussion surrounding the impacts of climate change raises significant questions regarding the enduring viability of human existence and the earth's ecological system. Supposing that our perspective on the future of human civilization and the planet is inherently gloomy, how does this affect a feeling of hope or despair for future generations, or influence our sense of personal and social significance and direction?

In this opinion paper, the authors discuss the impact of the climate crisis in the field of mental health, which has generated widespread clusters of eco-emotions and so-called “psychoterratic” syndromes (i.e., Earth-related mental syndromes) (Ágoston et al., 2022; Cianconi et al., 2023; Stanley et al., 2021). We then consider how ecological hope can be seen as the current manifestation of the responsibility and reconciliation between humans and our common home: earth. Restoring habitats and biological communities is urgent, and we must foster both the wellbeing of the earth and humanity through ecological restoration. We end by outlining practical steps to promote ecological hope in education for sustainable development.

## 2 Health and climate change

With the increasing impact of the ecological crisis and the public's growing awareness, there is a rising feeling of loss (including grief) over the species being pushed into extinction by modern industrial extraction and waste processes (Britnell et al., 2023). The primary causes of extinction include hunting, fishing, and altering habitats for agricultural, urban, and industrial purposes. However, species are also facing a growing threat of extinction due to anthropogenic climate change. This phenomenon is causing a rise in temperatures and extreme weather events in various biomes, leading to widespread mortality among species (Magnan et al., 2021). Likewise, economic instability, cultural erosion, and concerns about future consequences are all factors that contribute to mental health difficulties such as mood disorders, post traumatic stress disorder and increased substance addiction and suicide rate (Cianconi et al., 2020; Cuijpers et al., 2023). Simultaneously, major corporations and government agencies persist in pursuing a growth and development strategy that adheres to the status quo, relying on the continued extraction of fossil fuels and the conversion of forests and savannahs into agricultural and industrial areas.

The influence of rampant climate change on human health is intricately connected to several social, economic, and infrastructural issues that worsen disparities among different populations (Marujo, 2023). Climate change has unquestionably substantial effects on physical health, leading to around 12.6 million avoidable deaths annually due to environmental alterations (World Health Organization, 2024). Moreover, current research suggests that human-caused climate change also has adverse consequences on mental health (Walinski et al., 2023). Research suggests that people exposed to severe weather events, which are directly influenced by climate change, may experience detrimental mental health symptoms that significantly affect their overall wellbeing. Estimates indicate that 25–50% of these individuals are likely to develop such symptoms (Trombley et al., 2017).

Studies on the health consequences of climate change have primarily concentrated on the direct physical effects, specifically: fatalities and injuries caused by extreme weather events; the effects of rising temperatures and heat waves; the transmission of diseases by vectors; the impact on air quality and respiratory illnesses; and alterations in the quality and availability of food and water (Ebi et al., 2021; Rocque et al., 2021). Currently, other consequences of climate change are expected to have immediate effects on the frequency and intensity of mental health problems in impacted areas, as well as significant consequences for mental health services (Charlson et al., 2021; Li et al., 2022). Climate change has significant implications for mental health generated by concerns over personal wellbeing and security, exposing individuals to climate-related dysthymia (Romanello et al., 2023), eco-emotions and “psychoterratic” syndromes such as eco-anxiety, climate change anxiety, ecological grief, ecological stress, eco-paralysis (i.e., characterized by the inability to meaningfully respond to climatic and ecological challenges), environmental distress, and solastalgia (i.e., the nostalgia, anxiety, stress, and worry of people living in degraded environments) (Ágoston et al., 2022; Cianconi et al., 2023; Palinkas and Wong, 2020; Ramadan et al., 2023; Teo et al., 2024). Furthermore, susceptible populations are starting to encounter disturbances to the social, economic, and environmental factors that foster mental wellbeing (Crane et al., 2022). In parallel, there is a growing recognition that the distribution of health effects would disproportionately affect low-income or otherwise more susceptible people (Gianfredi et al., 2024; White et al., 2023).

Research has shown that the relationship with nature and the fear of a non-sustainable future are related with hope (Krafft et al., 2023). Likewise, mental health promotion is closely interconnected with individual and community hope, and with climate change in the long term (Newberry Le Vay et al., 2024). To mitigate an individual and a community's pessimism regarding the future, a pragmatic comprehension of the implications of climate change and the potential actions that can be taken is essential. Considering the increasingly dire predictions, people engaged in mental health promotion need to carefully consider the connection between evidence, hope, and action. The difficulties associated with adapting to climate change can inspire innovative ideas and actions that enhance the resilience and ingenuity of individuals and communities (Salvador Costa et al., 2022). Communities can promote cooperative efforts, both at the local level and on a global scale. In a recent scoping review, several mental health and psychosocial interventions were identified that operate at many levels, including individuals, groups, local media and institutions, and larger social structures (Xue et al., 2024). These interventions included a range of mechanisms of action such as “psychotherapy, resilience-building programs, nature-based activities, community strengthening networks, and climate activism projects” (Xue et al., 2024, p. 1). Furthermore, Seligman's (2011) research in positive psychology—examining the correlation between mental wellbeing, hope, and optimism—offers compelling evidence for the possibility of personal development and change arising from the climate issue.

## 3 Ecological hope: *Homo esperans* commitment to our common home

In the face of societal and ecological damage, we must actively seek hope and motivation to pursue a more promising future. In 2022, UN Secretary-General Guterres characterized the most recent report from the Intergovernmental Panel on Climate Change as an “atlas of human suffering” (United Nations, 2022). This report predicts that the prolonged climate crisis will generate injustices and growing vulnerabilities in our societies. Persistent colonialism, systemic racism, environmental injustice, as well as recent global emergencies (such as the COVID-19 pandemic, drought, and bushfires) serve as daily reminders of the perpetual instability we face (Deivanayagam et al., 2023; Marujo, 2023). To successfully navigate the problems of both the present and the future, we must be able to adapt. Hope is crucial in maintaining adaptability in the face of unpredictable social-ecological futures (Ojala, 2012; Wettergren, 2024), allowing for positive responses. People known as *Homo esperans* have an innate curiosity and actively engage in the exploration of the future, striving to establish ambitious objectives that they will ultimately achieve (Osika, 2019). Ecological hope arises from actively participating in activities that embody the fundamental ecological principle, expressed in Pope Francis' Encyclical Letter *Laudato Si': On Care for Our Common Home*, that all living beings are interconnected (Pope Francis, 2015).

Ecological hope as a dynamic multifaceted cognitive-emotional-motivational state that sensitizes individuals to the potential of desirable future outcomes has prompted many climate advocates and researchers to view hope as a predictor of climate action and engagement (Geiger et al., 2023). Following a transdisciplinary and culturally sensitive approach, hope is conceptualized as a wish or desire for a valued outcome or state of affairs, together with the belief that its achievement is feasible (although uncertain and not necessarily probable) and trust in the presence of internal or external resources that can help in its achievement, particularly when facing challenges and setbacks (Krafft et al., 2023). The literature explores several expressions of hope that are relevant to the social-ecological complexity we currently encounter (Bender and Rawluk, 2023). However, the organization of these expressions of hope varies. O'Hara (2014, p. 1) divided hope into three overarching categories: “particularized, generalized, and transformative expressions of hope”. Particularized hope centers around a particular objective or issue, such as reducing the impact of or adjusting to climate change, while maintaining an optimistic anticipatory and emotional state regarding the future (Ojala, 2017). Particularized hope succeeds when there is logical reasoning and individuals can act and control the situation. However, it offers little support when logic fails to provide solutions and when events and situations are beyond our control. Hope can also be generalized, focusing on a more abstract objective for positive outcomes at various levels (Havel, 2014), such as thriving or maintaining a sustainable lifestyle. Therefore, generalized hope is most effective when there is uncertainty about the way forward. Transformative hope arises when both generalized and particularized hope are combined, typically during a personal existential crisis, such as when an individual challenges the purpose of their life (Bender and Rawluk, 2023; O'Hara, 2014; Pleeging et al., 2022). In this vein, active contemplation and critical reflection lead to transformative hope, which allows individuals to shift their worldview and find contentment, regardless of the outcome (O'Hara, 2014). While transformative hope is also considered to critically negate the present, the focus of this form of hope is goal-directed social praxis. The process of envisioning an alternative future is meant to inspire action to create what is dreamt of Strazds (2019). A deeper self-awareness and awareness of otherness results in what Renaud (2023) calls the moral and ethical imperative of human responsibility to protect “Our common Home”. Each of these various expressions of hope can contribute to positive responses to social-ecological concerns.

Another aspect of hope that is being discussed is how individuals put hope into action. Certain literary works disparage passive hope, implying that it is synonymous with “false” hope (Osika, 2019). Passive hope is commonly linked to times of inactivity and a lack of agency or motivation (Lybbert and Wydick, 2018; Musschenga, 2019). The criticism of false hope primarily pertains to hope that is rooted in denial, and the mischaracterization of hope as mere optimism. Passive hope can be defined as the act of patiently awaiting improvement or avoiding damage. In this sense, individuals who label themselves as “doomsters” tend to disregard political and technological solutions to climate change as mere “hopium” (Doig, 2023). For Andersson (2016), passive waiting is a talent that can help individuals withstand a crisis. It acknowledges that even passive hope can provide internal support for individuals facing a catastrophe that might otherwise be psychologically overwhelming.

On the other hand, active hope suggests an engaged and observable activity (Orr, 2011). For de Graaf (2016), active hope involves a “temporal orientation of intentional and ethical action” (p. 604) and includes four components: visioning, expectations of results, emotions, and enacting. When envisioning hope, a systematic approach entails confronting the truth, identifying barriers and possibilities, considering interpersonal connections, and acknowledging the additional resources at hand (Diaz, 2024). It involves contemplating objectives, intentions, or values, evaluating an individual's capabilities and influence, identifying strategies to accomplish goals (Laranjeira and Querido, 2022), and determining methods of action (Osika, 2019).

The presence of both active and passive hope illustrates the flexible and responsive character of hope in relation to shifts in circumstances. Whether an individual or community possesses passive or active hope does not prevent them from also experiencing generalized, particularized, or transformative hope, as they can demonstrate various forms of hope in either a passive or active manner (Bender and Rawluk, 2023). By acknowledging the wide range of ways hope can be expressed, we can better understand how individuals might effectively respond to social and ecological changes that necessitate hope, particularly when taking action is crucial.

## 4 A pedagogy for a sustainable future

To effectively address social-ecological change, the concept of hope needs to be consistent with the underlying principles of social-ecological systems (SES) thinking, which understand the connections and interdependence between humans and nature. For instance, incorporation of learning and critical reflection is crucial in the governance and adaptive ecomanagement of SES. It is important to carefully consider the individuals who are engaged in the learning process (Li and Monroe, 2019), the connections between these learners, and the presence of conflicts and power imbalances within the social environment.

Development education and global learning can significantly contribute to global agendas on climate change, especially when incorporating the principles of Paulo Freire and his concept of the pedagogy of hope (Bourn, 2021; Irwin, 2012). When based on practical concerns and obstacles, hope can serve as a significant method for understanding global issues, despite its frequently perceived idealistic and utopian nature. Pedagogies of hope can both preserve and perpetuate current social relationships, as well as alter and change them (Finnegan and d'Abreu, 2024). Scholars have been studying the significance of incorporating climate hope into climate change education as a response to concerns about climate anxiety and distress. The Hope Wheel model (Finnegan and d'Abreu, 2024) questions the prevailing narratives of society and encourages the rejection of the underlying teachings that promote unsustainability. Instead, it promotes a pedagogy of hope that emphasizes the importance of relationships. This model involves three components: (a) a critical reasoning element that allows for the exploration of bold and disruptive questions regarding the reasons for the current state of affairs and the potential for change, as well as an examination of disinformation and misinformation; (b) a relational element serves as a connection between one's personal, inner self and other individuals who are not physically present or have different perspectives, as well as the non-human world; and, (c) an emancipatory element that emphasizes the ability to act independently and make decisions for oneself (Finnegan and d'Abreu, 2024).

A pedagogy of hope encourages educators to deliberately create opportunities for “transgressive learning”, provoking a transformation in the learner's perception of the world (Finnegan and d'Abreu, 2024; Frumkin, 2022). Participatory education methods like digital storytelling, peer-to-peer learning, self-awareness soft skills, and nature connectedness activities (Edwards et al., 2023) can address the well-documented worries and unease among young individuals regarding climate issues. Educators must recognize and address the dark perspectives on the global future, known as “probable futures,” and also assist young people in envisioning alternative “preferable futures” (Hicks, 2014). In this sense, some essential tenets should be applied to promote ecological hope in education for sustainable development (see Table 1). This can be achieved by using hope-driven, action-focused methods that protect learners' mental health and foster their ability to take initiative through innovation, collaboration, and a supportive environment.

**Table 1:** Essential tenets to promote ecological hope in education (Diaz, 2024; Hicks, 2014, 2018; Verlie et al., 2021).

•Sharing: establishing environments where young individuals can openly express their emotions about climate change, free from the fear of judgment or ridicule.
•Listening: attentively and genuinely paying attention to learners' thoughts and opinions, ensuring they feel properly engaged and convinced their contributions are accepted.
•Understanding: grasping the essence of climate change, its sources, effects, and outcomes, as well as the measures being implemented to reduce and adjust to it.
•Acting: understanding the tasks that need to be accomplished in various settings, such as the classroom, home, and community. Requires identifying individuals with whom one may collaborate and get continuous assistance.
•Solidarity: forming strong connections with people, the sense of unity and cooperation in working together as a team, and the feeling of fulfillment and encouragement from achieving common goals, are fundamental sources of ecological hope.
•Empathy: being surrounded by encouraging persons cultivates an optimistic worldview. Nevertheless, it is crucial to maintain a balance and exhibit empathy toward everyone, including those who have divergent viewpoints.
•Focus on the present: instead of fixating on the past or being anxious about the future, optimistic individuals maintain their focus on the current moment. Their main focus is on constant learning, gratitude, and self-improvement.
•Climate activism: evidence indicates that engaging in such activity has a positive impact on fostering hope, as well as alleviating feelings of fear and hopelessness (Frumkin, 2022). There is a probable positive feedback loop: “Activism fosters hope, and hope fosters activism” (Nabi et al., 2018).
•Decisiveness: empowering learners to enhance their decision-making skills, whether in relation to environmental concerns, social challenges, or personal aspirations.
•Self-awareness: acknowledging grief of a cherished location or the entire planet (“solastalgia” or “ecological grief”) can provide a feeling of peace, hope, and a greater will and ability to take action.

## 5 Final remarks

Overall, ecological hope as both a concept and a practical approach, is essential for navigating social-ecological transformations and co-creating embedded learning communities. To effectively convey social-ecological changes, we must possess a sense of hope, recognized for its role in fostering resilience, facilitating societal transformation, nurturing wellbeing and cultivating a belief in better futures. Nevertheless, various manifestations of hope can be unified to navigate intricate social-ecological transformations.

Critical reflection, relationality, and emancipation serve as the foundation for ecological hope, enabling us to acquire knowledge, undergo transformation, and react distinctly, including exploring different forms of hope. To implement ecological hope, it is necessary to invest in empirical research on each of its elements and develop the necessary skills to put it into action. Studying climate change may cause unease, but the potential for significant change may lie within this discomfort. As educators, we can cultivate hope and provide skills to mitigate climate change by promoting ecological restoration and seeking harmonious reconciliation between humans and nature, which means searching for *Homo esperans*. As the ecological crisis grows, there is greater awareness of the importance of protecting the most vulnerable communities and species, which promotes the flourishing of human beings and the planet toward a more climate-friendly future. Currently, a more in-depth investigation of strategies that promote ecosystem management, as well as the encouragement of educational programs and environmental conservation projects, is understood as essential.

## References

[B1] Ágoston C. Urbán R. Nagy B. Csaba B. Kováry Z. Kovács K.. (2022). The psychological consequences of the ecological crisis: three new questionnaires to assess ECO-anxiety, ECO-guilt, and ecological grief. Clim. Risk. Manag. 37:100441. 10.1016/j.crm.2022.100441

[B2] Andersson P. (2016). The dynamics of hope and motivations in groups working on complex societal issues. Integ. Rev. 12, 4–31.

[B3] Bender H. Rawluk A. (2023). Adaptive hope: a process for social environmental change. Ecol. Soc. 28:14. 10.5751/ES-14099-28021438179152

[B4] Bourn D. (2021). Pedagogy of hope: global learning and the future of education. Int. J. Dev. Educ. Global Learn. 13, 65–78. 10.14324/IJDEGL.13.2.01

[B5] Britnell J. A. Zhu Y. Kerley G. I. H. Shultz S. (2023). Ecological marginalization is widespread and increases extinction risk in mammals. Proc. Natl. Acad. Sci. U. S. A. 120:e2205315120. 10.1073/pnas.2205315120 36623195 PMC9934025

[B6] Charlson F. Ali S. Benmarhnia T. Pearl M. Massazza A. Augustinavicius J.. (2021). Climate change and mental health: a scoping review. Int. J. Environ. Res. Public Health 18:4486. 10.3390/ijerph18094486 33922573 PMC8122895

[B7] Cianconi P. Betrò S. Janiri L. (2020). The impact of climate change on mental health: a systematic descriptive review. Front. Psychiatry 11:74. 10.3389/fpsyt.2020.00074 32210846 PMC7068211

[B8] Cianconi P. Hanife B. Grillo F. Betro,' S. Lesmana C. B. Janiri L. (2023). Eco-emotions and psychoterratic syndromes: reshaping mental health assessment under climate change. Yale J. Biol. Med. 96, 211–226. 10.59249/EARX2427 37396973 PMC10303262

[B9] Clayton S. (2021). Climate change and mental health. Curr. Environ. Health Rep. 8, 1–6. 10.1007/s40572-020-00303-3 33389625

[B10] Crane K. Li L. Subramanian P. Rovit E. Liu J. (2022). Climate change and mental health: a review of empirical evidence, mechanisms and implications. Atmosphere 13:2096. 10.3390/atmos13122096 37727770 PMC10508914

[B11] Cuijpers P. Miguel C. Ciharova M. Kumar M. Brander L. Kumar P.. (2023). Impact of climate events, pollution, and green spaces on mental health: an umbrella review of meta-analyses. Psychol. Med. 53, 638–653. 10.1017/S0033291722003890 36606450 PMC9975983

[B12] de Graaf S. (2016). The construction and use of hope within health-settings: recent developments in qualitative research and ethnographic studies. Sociol. Comp. 10, 603–612. 10.1111/soc4.12380

[B13] Deivanayagam T. English S. Hickel J. Bonifacio J. Guinto R. Hill K.. (2023). Envisioning environmental equity: climate change, health, and racial justice. Lancet 402, 64–78. 10.1016/S0140-6736(23)00919-4 37263280 PMC10415673

[B14] Diaz J. (2024). Active hope as a catalyst for mental and psychosocial health in climate-related disasters. Psychology 15, 634–644. 10.4236/psych.2024.155039

[B15] Doig T. (2023). Friday Essay: If the World's Systems Are “Already Cracking” Due to Climate Change, Is There a Post-Doom Silver Lining? Available at: http://theconversation.com/friday-essay-if-the-worlds-systems-are-already-cracking-due-to-climate-change-is-there-a-post-doom-silver-lining-213890 (accessed July 15, 2024).

[B16] Ebi K. L. Vanos J. Baldwin J. W. Bell J. E. Hondula D. M. Errett N. A.. (2021). Extreme weather and climate change: population health and health system implications. Annu. Rev. Public Health 42, 293–315. 10.1146/annurev-publhealth-012420-105026 33406378 PMC9013542

[B17] Edwards R. C. Larson B. M. H. Clayton S. (2023). Navigating eco-anxiety and eco-detachment: educators' strategies for raising environmental awareness given students' disconnection from nature. Environ. Educ. Res. 30, 864–880. 10.1080/13504622.2023.2286929

[B18] Finnegan W. d'Abreu C. (2024). The hope wheel: a model to enable hope-based pedagogy in Climate Change Education. Front. Psychol. 15:1347392. 10.3389/fpsyg.2024.1347392 38572209 PMC10987955

[B19] Frumkin H. (2022). Hope, health, and the climate crisis. J. Clim. Change Health 5:100115. 10.1016/j.joclim.2022.100115

[B20] Geiger N. Dwyer T. Swim J. K. (2023). Hopium or empowering hope? A meta-analysis of hope and climate engagement. Front. Psychol. 14:1139427. 10.3389/fpsyg.2023.1139427 37649687 PMC10465179

[B21] Gianfredi V. Mazziotta F. Clerici G. Astorri E. Oliani F. Cappellina M.. (2024). Climate change perception and mental health. Results from a systematic review of the literature. Eur. J. Investig. Health Psychol. 14, 215–229. 10.3390/ejihpe14010014 38248134 PMC10814599

[B22] Havel V. (2014). “An orientation of the heart,” in The Impossible Will Take a Little While: Perseverance and Hope in Troubled Times, ed. P. Loeb (New York, NY: Basic Books), 106–112.

[B23] Hicks D. (2014). Educating for Hope in Troubled Times: Climate change and the transition to a post-carbon future. London: Trentham Books.

[B24] Hicks D. (2018). Why we still need a geography of hope. Geography 103, 78–85. 10.1080/00167487.2018.12094041

[B25] Irwin J. (2012). Paulo Freire's Philosophy of Education. London: Continuum.

[B26] Krafft A. M. Guse T. Slezackova A. (2023). “Theoretical foundations and a transdisciplinary concept of hope,” in Hope across cultures. Cross-Cultural Advancements in Positive Psychology, Vol 14, eds. A. M. Krafft, T. Guse, and A, Slezackova (Cham: Springer).

[B27] Laranjeira C. Querido A. (2022). Hope and optimism as an opportunity to improve the “positive mental health” demand. Front. Psychol. 13:827320. 10.3389/fpsyg.2022.827320 35282230 PMC8907849

[B28] Li C. Lawrance E. L. Morgan G. Brown R. Greaves N. Krzanowski J.. (2022). The role of mental health professionals in the climate crisis: an urgent call to action. Int. Rev. Psychiatry 34, 563–570. 10.1080/09540261.2022.2097005 36165755

[B29] Li C. J. Monroe M. C. (2019). Exploring the essential psychological factors in fostering hope concerning climate change. Environ. Educ. Res. 25, 936–954. 10.1080/13504622.2017.1367916

[B30] Lybbert T. Wydick B. (2018). Poverty, aspirations, and the economics of hope. Econ. Dev. Cult. Change 6, 709–753. 10.1086/696968

[B31] Magnan A. K. Pörtner H.-O. Duvat V. K. E. Garschagen M. Guinder V. A. Zommers Z.. (2021). Estimating the global risk of anthropogenic climate change. Nat. Clim. Change 11, 879–885. 10.1038/s41558-021-01156-w

[B32] Marujo H. Á. (2023). The nexus between peace and mental well-being: contributions for public happiness. Mental Health Soc. Inclus. 27, 355–379. 10.1108/MHSI-07-2023-007738747358

[B33] Musschenga B. (2019). Is there a problem with false hope? J. Med. Philos. 44, 423–441. 10.1093/jmp/jhz010 31356659 PMC6900746

[B34] Nabi R. L. Gustafson A. Jensen R. (2018). Framing climate change: exploring the role of emotion in generating advocacy behavior. Sci. Commun. 40, 442–468. 10.1177/1075547018776019

[B35] Newberry Le Vay J. Cunningham A. Soul L. Dave H. Hoath L. Lawrance E. L. (2024). Integrating mental health into climate change education to inspire climate action while safeguarding mental health. Front. Psychol. 14:1298623. 10.3389/fpsyg.2023.1298623 38259528 PMC10800611

[B36] O'Hara D. (2014). “Three spheres of hope: generalised, particularised, and transformative,” in Phoenix Rising From Contemporary Global Society. eds. L. Ortiz, and D. O'Hara (Leiden: Brill), 1–14.

[B37] Ojala M. (2012). Hope and climate change: importance of hope for environmental engagement among young people. Environ. Educ. Res. 18, 625–542. 10.1080/13504622.2011.63715727409075

[B38] Ojala M. (2017). Hope and anticipation in education for a sustainable future. Futures 94, 76–84. 10.1016/j.futures.2016.10.004

[B39] Orr D. (2011). Hope Is an Imperative: The Essential David Orr. Washington, DC: Island Press.

[B40] Osika G. (2019). On hoping for hope: the search for homo esperans. Ethos 32, 136–153.

[B41] Palinkas L. A. Wong M. (2020). Global climate change and mental health. Curr. Opin. Psychol. 32, 12–16. 10.1016/j.copsyc.2019.06.023 31349129

[B42] Pleeging E. van Exel J. Burger M. (2022). Characterizing hope: an interdisciplinary overview of the characteristics of hope. Appl. Res. Qual. Life 17, 1681–1723. 10.1007/s11482-021-09967-x27409075

[B43] Pope Francis (2015). Laudato Si': On Care for Our Common Home. Rome: Vatican Press.

[B44] Ramadan R. Randell A. Lavoie S. Gao C. Manrique P. C. Anderson R.. (2023). Empirical evidence for climate concerns, negative emotions and climate-related mental ill-health in young people: a scoping review. Early Interv. Psychiatry 17, 537–563. 10.1111/eip.13374 36641809

[B45] Renaud M. (2023). “Human responsibility for the protection of our “common home”,” in Blue Planet Law. Sustainable Development Goals Series, eds. A. G. Garcia, and A, Cortês (Cham: Springer).33505035

[B46] Rocque R. J. Beaudoin C. Ndjaboue R. Cameron L. Poirier-Bergeron L. Poulin-Rheault R. A.. (2021). Health effects of climate change: an overview of systematic reviews. BMJ Open 11:e046333. 10.1136/bmjopen-2020-046333 34108165 PMC8191619

[B47] Romanello M. Napoli C. D. Green C. Kennard H. Lampard P. Scamman D.. (2023). The 2023 report of the Lancet Countdown on health and climate change: the imperative for a health-centred response in a world facing irreversible harms. Lancet 402, 2346–2394. 10.1016/S0140-6736(23)01859-7 37977174 PMC7616810

[B48] Salvador Costa M. J. Leitão A. Silva R. Monteiro V. Melo P. (2022). Climate change prevention through community actions and empowerment: a scoping review. Int. J. Environ. Res. Public Health 19:14645. 10.3390/ijerph192214645 36429383 PMC9690446

[B49] Seligman M. (2011). Learned Optimism. London: William Heinemann.

[B50] Stanley S. Hogg T. Leviston Z. Walker I. (2021). From anger to action: differential impacts of eco-anxiety, eco-depression, and eco-anger on climate action and wellbeing. J. Clim. Change Health 1:100003. 10.1016/j.joclim.2021.100003

[B51] Strazds L. (2019). Radical hope: transforming sustainability. J. Sustain. Educ. 21, 1–18.

[B52] Teo S. Gao C. Brennan N. Fava N. Simmons M. Baker D.. (2024). Climate change concerns impact on young Australians' psychological distress and outlook for the future. J. Environ. Psychol. 93, 1–11. 10.1016/j.jenvp.2023.102209

[B53] Trombley J. Chalupka S. Anderko L. (2017). Climate change and mental health. Am. J. Nurs. 117, 44–52. 10.1097/01.NAJ.0000515232.51795.fa 28333743

[B54] United Nations (2022). Remarks by António Guterres, Secretary-General of the United Nations, to the Press Conference Launch of IPCC Report. Available at: https://media.un.org/en/asset/k1x/k1xcijxjhp#:~:text=Today's IPCC report is an,in the danger zone-now (accessed July 15, 2024).

[B55] Verlie B. Clark E. Jarrett T. Supriyono E. (2021). Educators' experiences and strategies for responding to ecological distress. Aust. J. Environ. Educ. 37, 132–146. 10.1017/aee.2020.3431933159

[B56] Walinski A. Sander J. Gerlinger G. Clemens V. Meyer-Lindenberg A. Heinz A. (2023). The effects of climate change on mental health. Dtsch. Arztebl. Int. 120, 117–124. 10.3238/arztebl.m2022.0403 36647584 PMC10154789

[B57] Wettergren Å. (2024). Emotionalising Hope in Times of Climate Change. Emotions and Society, 1–19. Available at: https://bristoluniversitypressdigital.com/view/journals/emsoc/aop/article-10.1332-26316897Y2024D000000021/article-10.1332-26316897Y2024D000000021.xml (accessed July 15, 2024).

[B58] White B. P. Breakey S. Brown M. J. Smith J. R. Tarbet A. Nicholas P. K.. (2023). Mental health impacts of climate change among vulnerable populations globally: an integrative review. Ann. Global Health 89:66. 10.5334/aogh.4105 37810609 PMC10558031

[B59] World Health Organization (2024). Climate Change. Available at: https://www.who.int/health-topics/climate-change (accessed July 15, 2024).

[B60] Xue S. Massazza A. Akhter-Khan S. C. Wray B. Husain M. I. Lawrance E. L. (2024). Mental health and psychosocial interventions in the context of climate change: a scoping review. NPJ Mental Health Res. 3:10. 10.1038/s44184-024-00054-1 38609540 PMC10956015

